# Community and Patient Features and Health Care Point of Entry for Pediatric Concussion

**DOI:** 10.1001/jamanetworkopen.2024.42332

**Published:** 2024-10-30

**Authors:** Daniel J. Corwin, Daniele Fedonni, Catherine C. McDonald, Alexis Peterson, Juliet Haarbauer-Krupa, Melissa Godfrey, Peter Camacho, Tyra Bryant-Stephens, Christina L. Master, Kristy B. Arbogast

**Affiliations:** 1 Division of Emergency Medicine, Children’s Hospital of Philadelphia, Philadelphia, Pennsylvania; 2 Center for Injury Research and Prevention, Children’s Hospital of Philadelphia, Philadelphia, Pennsylvania; 3 Perelman School of Medicine at the University of Pennsylvania, Philadelphia; 4 Department of Biomedical and Health Informatics, Children’s Hospital of Philadelphia, Philadelphia, Pennsylvania; 5 Department of Family and Community Health, University of Pennsylvania School of Nursing, Philadelphia; 6 US Centers for Disease Control and Prevention, Atlanta, Georgia; 7 Division of General Pediatrics, Children’s Hospital of Philadelphia, Philadelphia, Pennsylvania; 8 Sports Medicine and Performance Center, Children’s Hospital of Philadelphia, Philadelphia, Pennsylvania

## Abstract

**Importance:**

Many recent advances in pediatric concussion care are implemented by specialists; however, children with concussion receive care across varied locations. Thus, it is critical to identify which children have access to the most up-to-date treatment strategies.

**Objective:**

To evaluate differences in the sociodemographic and community characteristics of pediatric patients who sought care for concussion across various points of entry into a regional health care network.

**Design, Setting, and Participants:**

This cross-sectional study included children seen for concussions across a regional US health care network from January 1, 2017, to August 4, 2023. Pediatric patients aged 0 to 18 years who received an *International Statistical Classification of Diseases and Related Health Problems, Tenth Revision, Clinical Modification *code for concussion were included. The study took place at emergency department (ED) and outpatient (primary care [PC] and specialty care [SC]) settings.

**Exposures:**

Age at visit, biological sex, parent-identified race and ethnicity, payer type, median income and percentage of adults with a bachelor’s degree for home zip code, and overall and subdomain Child Opportunity Index (COI) score based on patient address.

**Main Outcomes and Measures:**

The association of exposures with point of entry of ED, PC, and SC were examined in both bivariate analysis and a multinomial logistic regression.

**Results:**

Overall, 15 631 patients were included in the study (median [IQR] age, 13 [11-15] years; 7879 [50.4%] male; 1055 [6.7%] Hispanic, 2865 [18.3%] non-Hispanic Black, and 9887 [63.7%] non-Hispanic White individuals). Race and ethnicity were significantly different across settings (1485 patients [50.0%] seen in the ED were non-Hispanic Black vs 1012 [12.0%] in PC and 368 [8.7%] in SC; *P* < .001) as was insurance status (1562 patients [52.6%] seen in the ED possessed public insurance vs 1624 [19.3%] in PC and 683 [16.1%] in SC; *P* < .001). Overall and individual COI subdomain scores were also significantly different between settings (overall COI median [IQR]: ED, 30 [9-71]; PC, 87 [68-95]; SC, 87 [69-95]; *P* < .001). Race, insurance status, and overall COI had the strongest associations with point of entry in the multivariable model (eg, non-Hispanic Black patients seen in the ED compared with non-Hispanic White patients: odds ratio, 2.03; 95% CI, 1.69-2.45).

**Conclusions and Relevance:**

In this cross-sectional study, children with concussion seen in the ED setting were more likely to be non-Hispanic Black, have public insurance, and have a lower Child Opportunity Index compared with children cared for in the PC or SC setting. This highlights the importance of providing education and training for ED clinicians as well as establishing up-to-date community-level resources to optimize care delivery for pediatric patients with concussion at high risk of care inequities.

## Key Points

**Question:**

Are community and sociodemographic characteristics associated with the location of initial point of entry into the health care system for pediatric patients with concussion?

**Findings:**

In this cross-sectional study of 15 631 pediatric patients with concussion, there were significant differences in the proportions of non-Hispanic Black patients (50.0% vs 12.0% vs 8.7%) and those with public insurance (52.6% vs 19.3% vs 16.1%) who sought care in the emergency department compared with primary and specialty care settings. There were also differences in Child Opportunity Index scores among children seen in the emergency departments vs other settings.

**Meaning:**

These findings suggest that specific education and training for pediatric emergency medicine clinicians and establishing up-to-date community-level resources are critical to optimize equitable concussion care delivery.

## Teaser

This cross-sectional study analyzes whether sociodemographic and community characteristics are associated with the health care setting in which pediatric patients with concussion receive initial care.

## Introduction

Concussion is a common injury in children and adolescents.^1,2^ Historically, management of concussion has focused primarily on passive rest and observation.^3^ Over the past decade, several advances in concussion management have occurred, with emerging evidence demonstrating the efficacy of active, individualized rehabilitation strategies.^4,5,6,7^ While these interventions represent important advances, they are almost exclusively recommended by pediatric concussion specialists.^8^ However, previous work has shown that most pediatric patients with concussion are cared for outside of the specialty setting.^9^ In addition, access to concussion specialists is not evenly distributed across sociodemographic groups. Prior work has shown that, when compared with those with musculoskeletal injuries, Hispanic patients, non–English-speaking patients, and those with public insurance are less likely to seek care for concussion from a specialist.^10^ Disparities for pediatric concussion extend beyond clinical settings, particularly in knowledge of concussion management and resource access. These include variability in willingness to report concussion symptoms across race,^11^ likelihood to receive a concussion diagnosis across race and ethnicity,^12^ access to resources available following injury across race,^13^ and ability to complete care recommendations across race and insurance status.^14^

While prior research from our group has evaluated demographic differences for pediatric patients with concussion by point of entry into our health care system,^9^ often only race, ethnicity, and insurance status are used as proxies to characterize patients from a socioeconomic perspective, and this work predates knowledge of the more recent active management strategies. This limits an understanding of the community-level infrastructure that may serve as a facilitator or barrier to care. In addition, the concept of race itself is troublesome as a sole marker of disparities; race is a social concept, without biologic validity, and has historically been used to justify discrimination and social hierarchies. At best, it serves as a proxy for structural racism that can impede care delivery.^15^ In the past decade, the Child Opportunity Index (COI) has emerged as a robust composite measure to capture critical health opportunities available to children.^16^ While some work has been done evaluating outcome differences by COI for children sustaining severe traumatic brain injury,^17^ to date, no studies of which we are aware have evaluated differences that may exist across different points of entry into the health care system for pediatric concussion.

Therefore, the objective of this study was to use a registry of pediatric patients with concussion seen in different settings across our regional health care network to evaluate differences in individual- and neighborhood-level opportunity based on point of entry. We hypothesized that children seeking care in the specialty care (SC) and primary care (PC) settings would be less likely to be non-Hispanic Black, less likely to possess public insurance, and have higher average COI compared with those seeking care in the emergency department (ED).

## Methods

### Patient Population

In this cross-sectional study, data were collected and are reported in accordance with Strengthening the Reporting of Observational Studies in Epidemiology (STROBE).^18^ Data were obtained from the Minds Matter Concussion Registry. While thoroughly described elsewhere,^19,20,21^ it comprises electronic health record (EHR) information for children, adolescents, and young adults evaluated for possible head injury within our health care network, which encompasses inpatient and outpatient settings of a large, tertiary care children’s hospital network. This includes an ED (located in an urban setting), 31 PC locations, and 7 SC practices located across 2 states (both encompass urban and suburban areas, with varied accessibility by public transportation). For the current study, we limited the cohort to patients who were aged 0 to 18 years, who presented from January 1, 2017, through August 4, 2023, and who received an *International Statistical Classification of Diseases, Tenth Revision, Clinical Modification *(*ICD-10-CM*) diagnosis code of concussion. Only initial visits, defined as the first clinical encounter in the EHR, and only the first concussion for those who experienced multiple concussions across the study period were included. Given our reliance on demographic data and anticipated small degree of missingness, any patient with missing data (including address to calculate neighborhood characteristics) was excluded. This study was approved by the Children’s Hospital of Philadelphia’s institutional review board, from whom a waiver of informed consent was granted because obtaining consent would be infeasible, data were deidentified, and the study posed no more than minimal risk to participants.

### Variables and Outcomes

The registry includes clinical and demographic variables extracted from the EHR.^19,22^ For the current analysis, we used the following data elements: age at visit; biological sex (as reported by patient or family); race and ethnicity (provided as 2 separate variables at the time of registration, directly by patient and family or entered by a registration staff member); payer type (based on insurance information from the EHR); median income and percentage of adults age 25 years or older with a bachelor’s degree within the zip code of patient’s home address at the time of visit (determined by the 2020 American Community Survey 5-Year Estimates)^23^; and COI domains from the COI version 3.0 (as determined by census tract information for the patient’s home address at the time of visit).^16^ Although race and ethnicity are dynamic, social constructs, and the sampled categories do not represent underlying physiologic differences, to align with commonly established categories in the United States as well as previous categorizations of care-seeking behavior by pediatric patients with concussion, we collapsed race and ethnicity into the following variables: Hispanic, non-Hispanic Black, non-Hispanic White, and other (including American Indian or Alaska Native, Asian, Native Hawaiian or Other Pacific Islander, and multiple races).^9,12^ Payer type was coded as either public or private insurance; none were coded as uninsured, as those with missing insurance data, which potentially included uninsured patients (<1% of sample), were excluded. The nationally normative score for the overall COI, as well as the 3 COI subdomains (social and economic, educational, and health and environment), were generated based on the patient’s address; COI scores range from 0 to 100, where higher scores represent a higher degree of opportunity.^17,18^

### Statistical Analysis

We used standard descriptive statistics to describe overall sociodemographic and zip code and census tract characteristics. Categorical variables were compared across clinical setting (ED, PC, and SC) by Pearson χ^2^ tests, and continuous variables were compared by Kruskal-Wallis rank sum tests.

Following bivariate analyses, the associations of health care setting with individual- and neighborhood-level characteristics were analyzed using a multinomial logistic regression to further elucidate the variables most strongly associated with point of entry, using SC as the reference setting, as it generally provides the most-up-to-date care strategies. Within the regression, odds for median income and COI were presented for each $10 000 and 10-point increase, respectively, to allow for better interpretability; 95% CIs were estimated using standard errors from the multinomial logistic regression model.^24^

Finally, to assess for potential impact of the COVID-19 pandemic on care-seeking behavior, which has been demonstrated across prior studies to have affected presentation to care,^25,26^ we performed a sensitivity analysis dividing our sample into 3 time periods: pre–COVID-19 (prior to March 16, 2020, when all nonessential businesses in the county of the primary hospital were closed), early COVID-19 (March 16, 2020, through March 15, 2021), and late COVID-19 (March 16, 2021, through the end of the study period).^27^ Statistical analyses were performed using R version 4.0.3 (R Project for Statistical Computing). Statistical significance was set at α = .05.

## Results

**Figure 1.**
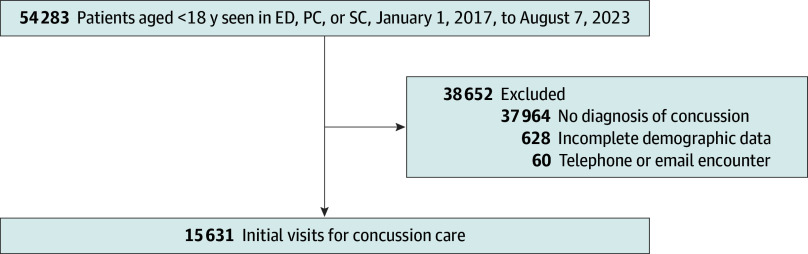
Flow Diagram of Study Population All visits for head trauma across the regional care network are included in the registry. For the current study, only those patients who were seen in the emergency department (ED), primary care (PC), or concussion specialty care (SC) for in-person visits, with a concussion diagnosis, and with complete demographic data were included.

Of the 15 631 patients (7879 [50.4%] male; 1055 [6.7%] Hispanic, 2865 [18.3%] non-Hispanic Black, and 9887 [63.7%] non-Hispanic White individuals) diagnosed with a concussion across the study period (Figure 1), 4245 (27.2%) were initially seen in a SC setting, 8417 (53.8%) in a PC setting, and 2969 (19.0%) in the ED. In total, 628 patients were excluded from the original sample due to missing data. Overall characteristics for the sample are presented in Table 1.

**Table 1.** Selected Characteristics of Initial Concussion Visit Among 15 631 Pediatric Patients

Characteristic	Patients, No. (%) (N = 15 631)
Location	
Emergency department	2969 (19.0)
Primary care	8417 (53.8)
Specialty care	4245 (27.2)
Age, y	
0-4.99	471 (3.0)
5.00-7.99	991 (6.3)
8.00-12.99	4774 (30.5)
13.00-17.99	9395 (60.1)
Biological sex	
Female	7752 (49.6)
Male	7879 (50.4)
Race and ethnicity	
Hispanic	1055 (6.7)
Non-Hispanic Black	2865 (18.3)
Non-Hispanic White	9887 (63.3)
Other^a^	1824 (11.7)
Insurance type	
Private	11 762 (75.2)
Public	3869 (24.8)
Income of residential zip code, median (IQR), US $	87 216 (70 827-108 674)
Residents in patient’s zip code with bachelor’s degree or higher, median (IQR), %	45 (29-57)
Child Opportunity Index, median (IQR)^b^	
Overall	83 (55-94)
Educational	84 (50-95)
Health and environmental	77 (56-90)
Social and economic	81 (52-92)

^a^ Other includes American Indian or Alaska Native, Asian, and Native Hawaiian or Other Pacific Islander children as well as multiracial children, children for whom race was not reported, and those who selected other race during registration.

^b^ Child Opportunity Index version 3.0 is a scaled *z* score that ranges from 0 to 100, where 0 represents the least opportunity and 100 represents the most opportunity.

**Figure 2.**
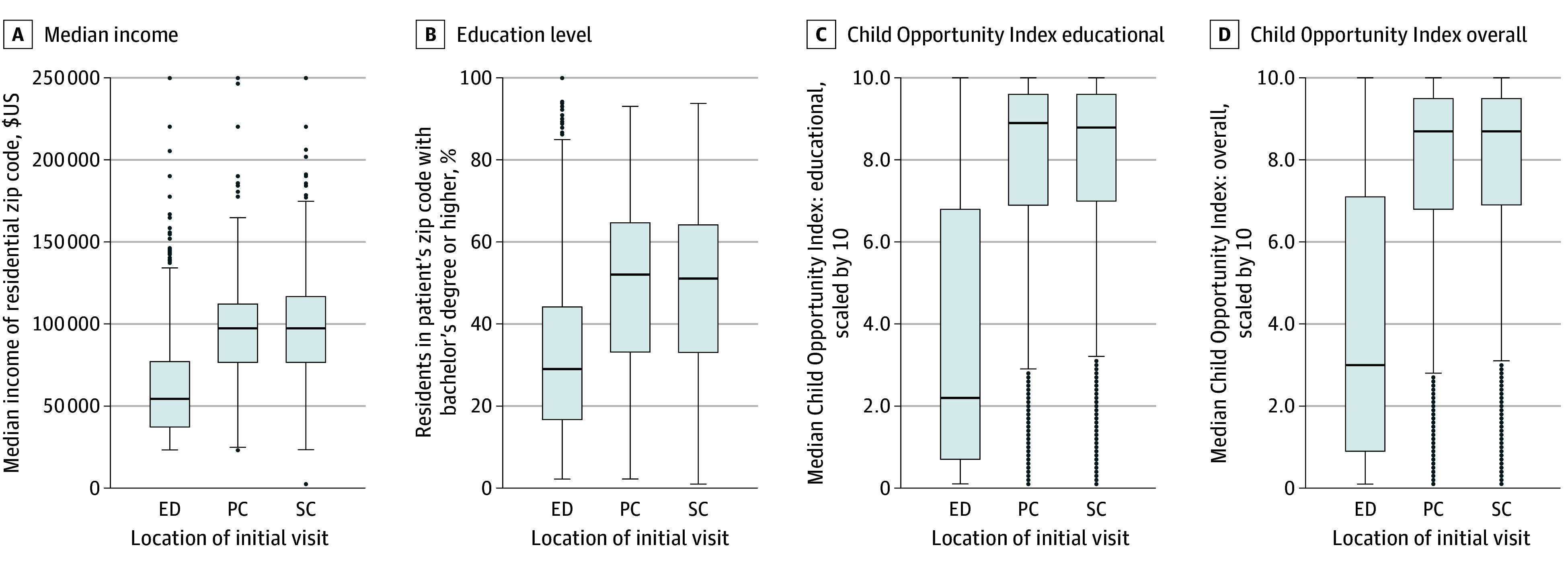
Distribution of Neighborhood Characteristics Across Initial Visit Location The Child Opportunity Index version 3.0 is a scaled *z* score that ranges from 0 to 100, where 0 represents the least opportunity and 100 represents the most opportunity. Center lines represent medians, with box edges indicating the IQR. Whiskers extend to the standard median × 1.5, with values outside that range indicated by dots. ED indicates emergency department; PC, primary care; and SC, specialty care.

We found significant differences among sociodemographic and zip code– or census tract–level characteristics across clinical settings (Table 2 and Figure 2). Specifically, age was significantly different across sites (<13 years: ED, 1675 patients [56.4%]; PC, 3403 [40.4%]; SC 1158 [27.3%]; *P* < .001) as were race (1485 patients [50.0%] seen in the ED were non-Hispanic Black vs 1012 [12.0%] in PC and 368 [8.7%] in SC; *P* < .001) and insurance status (1562 patients [52.6%] seen in the ED possessed public insurance vs 1624 [19.3%] in PC and 683 [16.1%] in SC; *P* < .001). Differences were also seen in median income and bachelor’s degree possession. Finally, there were significant differences in COI. The median (IQR) COI for the ED was 30 (9-71) vs 87 (68-95) for PC and 87 (69-95) for SC (*P* < .001), with similar differences for each subdomain.

**Table 2.** Selected Characteristics of Initial Concussion Visit by Presenting Location

Characteristic	Patients, No. (%)			*P* value
	ED (n = 2969)	PC (n = 8417)	SC (n = 4245)	
Age, y				
0-4.99	287 (9.8)	140 (1.7)	44 (1.0)	<.001
5.00-7.99	370 (12.5)	485 (5.8)	136 (3.2)	
8.00-12.99	1018 (34.3)	2778 (33.0)	978 (23.0)	
13.00-17.99	1294 (43.6)	5014 (59.6)	3087 (72.7)	
Biological sex				
Female	1278 (43.0)	4148 (49.3)	2326 (54.8)	<.001
Male	1691 (57.0)	4269 (50.7)	1919 (45.2)	
Race and ethnicity				
Hispanic	247 (8.3)	534 (6.3)	274 (6.5)	<.001
Non-Hispanic Black	1485 (50.0)	1012 (12.0)	368 (8.7)	
Non-Hispanic White	944 (31.8)	5873 (69.8)	3070 (72.3)	
Other^a^	293 (9.9)	998 (11.9)	533 (12.6)	
Insurance type				
Private	1407 (47.4)	6793 (80.7)	3562 (83.9)	<.001
Public	1562 (52.6)	1624 (19.3)	683 (16.1)	
Income of zip code, median (IQR), US $	54 353 (37 434-77 091)	97 515 (76 854-112 373)	97 305 (76 710-116 895)	<.001
Residents in zip code with bachelor’s degree or higher, median (IQR), %	29 (18-42)	49 (33-60)	49 (33-60)	<.001
COI, median (IQR)^b^				
Overall	30 (9-71)	87 (68-95)	87 (69-95)	<.001
Educational	22 (7-68)	89 (69-96)	88 (70-96)	<.001
Health and environmental	58 (36-78)	79 (60-91)	81 (64-91)	<.001
Social and economic	30 (7-71)	87 (68-95)	87 (69-95)	<.001

Abbreviations: COI, Child Opportunity Index; ED, emergency department; PC, primary care; SC, specialty care.

^a^ Other includes American Indian or Alaska Native, Asian, Native Hawaiian or Other Pacific Islander children, as well as multiracial children, children for whom race was not reported, and those who selected “other race” during registration.

^b^ Child Opportunity Index version 3.0 is a scaled *z* score that ranges from 0 to 100, where 0 represents the least opportunity and 100 represents the most opportunity.

In the multivariable model (Table 3), compared with SC point of entry and their non-Hispanic White peers (referent group), non-Hispanic Black patients had greater odds of being seen in the ED (odds ratio [OR], 2.03; 95% CI, 1.69-2.45) or PC (OR, 1.56; 95% CI, 1.34-1.82). Compared with SC and those with private insurance (referent group), those with public insurance also had greater odds of being seen in the ED (OR, 1.65; 95% CI, 1.43-1.89) or PC (OR, 1.18; 95% CI, 1.06-1.31). Finally, compared with SC (referent group), each 10-point increase in overall COI was associated with lower odds of being seen in the ED (OR, 0.47; 95% CI, 0.31-0.70).

**Table 3.** Selected Characteristics and Location of Initial Visit for Concussion

Characteristic	OR (95% CI)^a^			
	Emergency department		Primary care	
	Univariate	Multivariable	Univariate	Multivariable
Age, y				
0-4.99	1 [Reference]	1 [Reference]	1 [Reference]	1 [Reference]
5.00-7.99	0.42 (0.29-0.61)^b^	0.37 (0.25-0.56)^b^	1.12 (0.76-1.65)	1.12 (0.76-1.66)
8.00-12.99	0.16 (0.11-0.22)^b^	0.16 (0.11-0.22)^b^	0.89 (0.63-1.26)	0.88 (0.62-1.25)
13.00-17.99	0.06 (0.05-0.09)^b^	0.07 (0.05-0.10)^b^	0.51 (0.36-0.72)^b^	0.51 (0.36-0.72)^b^
Biological sex				
Female	1 [Reference]	1 [Reference]	1 [Reference]	1 [Reference]
Male	1.60 (1.46-1.76)^b^	1.36 (1.21-1.52)^b^	1.25 (1.16-1.34)^b^	1.16 (1.08-1.26)^b^
Race and ethnicity				
Hispanic	2.93 (2.43-3.53)^b^	0.95 (0.77-1.19)	1.02 (0.88-1.19)	1.00 (0.86-1.18)
Non-Hispanic Black	13.10 (11.50-15.00)^b^	2.03 (1.69-2.45)^b^	1.44 (1.27-1.63)^b^	1.56 (1.34-1.82)^b^
Non-Hispanic White	1 [Reference]	1 [Reference]	1 [Reference]	1 [Reference]
Other^c^	1.79 (1.52-2.10)^b^	0.97 (0.81-1.17)	0.98 (0.87-1.10)	0.93 (0.83-1.05)
Insurance type				
Private	1 [Reference]	1 [Reference]	1 [Reference]	1 [Reference]
Public	5.79 (5.19-6.46)^b^	1.65 (1.43-1.89)^b^	1.25 (1.13-1.38)^b^	1.18 (1.06-1.31)^b^
Median income of ZIP code, per $10 000 increase	0.65 (0.64-0.66)^b^	0.75 (0.72-0.78)^b^	0.98 (0.97-1.00)	0.94 (0.92-0.96)^b^
Residents with bachelor’s degree or higher, %	0.95 (0.95-0.96)^b^	1.05 (1.04-1.05)^b^	1.00 (1.00-1.00)	1.01 (1.01-1.02)^b^
COI, per 10-point increase^d^				
Overall	0.67 (0.66-0.68)^b^	0.47 (0.31-0.70)^b^	0.99 (0.97-1.00)	0.75 (0.56-1.00)
Educational	0.69 (0.67-0.70)^b^	1.04 (0.91-1.18)^b^	1.00 (0.99-1.01)	1.17 (1.06-1.29)^b^
Health and environment	0.74 (0.72-0.75)^b^	1.11 (1.05-1.17)^b^	0.95 (0.93-0.96)^b^	0.93 (0.89-0.96)^b^
Social and economic	0.67 (0.66-0.68)^b^	1.49 (1.14-1.95)^b^	0.99 (0.97-1.00)	1.23 (1.02-1.49)^b^

Abbreviations: COI, child opportunity index; OR, odds ratio.

^a^ These results examine the association of factors with first visit occurring in the emergency department or primary care setting compared with the specialty care setting. For example, compared with specialty care point of entry and their non-Hispanic White peers, non-Hispanic Black patients had greater odds of being seen in the ED (OR, 2.03; 95% CI, 1.69-2.45) or PC (OR, 1.56; 95% CI, 1.34-1.82).

^b^ Statistically significant.

^c^ Other includes American Indian or Alaska Native, Asian, Native Hawaiian or Other Pacific Islander children as well as multiracial children, children for whom race as not reported, and those who selected “other race” during registration.

^d^ Child Opportunity Index is a scaled *z* score that ranges from 0 to 100, where 0 represents the least opportunity and 100 represents the most opportunity.

The results of our sensitivity analysis evaluating differences in care-seeking during the COVID pandemic are shown in the eTable in Supplement 1. Overall, we found that while similar differences existed during the first year of the pandemic compared with the overall sample, they were smaller in scale. Nearly all changes partially or fully reversed during the later pandemic time-period.

## Discussion

This study found key differences in point of entry into the health care system for pediatric patients with concussion across a large, regional, integrated health care network when examining community characteristics and patient sociodemographic features. Specifically, we found non-Hispanic Black patients, compared with non-Hispanic White patients, and those with public insurance, compared with those with private insurance, were more likely to initially seek concussion care in the ED. In addition, we found that those initially seeking concussion care in the ED were significantly more likely to have lower overall and individual subdomain COI scores compared with those seeking care in SC settings. These differences have important implications for the care of pediatric patients with concussion.

The differences in point of entry by age, race, ethnicity, and insurance are in line with previous work. A prior study of our regional health care network also found that non-Hispanic Black patients were more likely to first seek concussion care in the ED compared with their non-Hispanic White peers,^12^ as were pediatric patients with public insurance compared with those with private insurance and younger compared with older patients.^9^ Nationwide, once pediatric patients with concussion enter the health care system, it appears that inequities persist. Prior studies have demonstrated that Black children are less likely than White children to receive a concussion diagnosis when presenting to the ED following head trauma and are less likely than White children to receive emergent neuroimaging while in the ED.^28,29^ Such disparities extend to follow-up care; non-Hispanic Black children, in a study of children seen for concussion in PC, were less likely to complete care recommendations than non-Hispanic White and Hispanic peers, as were patients with public insurance.^14^ The etiology of these differences is likely multifactorial, including implicit biases among clinicians in making the concussion diagnosis (perhaps due to differing perceptions of pain, perceptions of symptom reporting, or level of suspicion for concussion),^30,31^ population-level knowledge disparities among racial groups (as studies have found differences across race in concussion knowledge and symptom recognition),^11,32^ and structural barriers that prevent Black children from seeking concussion care in the SC setting (eg, most of our SC settings are located in suburban locations). We must acknowledge, in spite of differences in this study and previous work, the limitations of utilizing race in this manner, given both its nature as a social construct without a biologic basis as well as the vast cultural and ethnic diversity that exists within our categories of race and ethnicity, all of which can impact care-seeking behavior. However they arise, these differences in concussion care-seeking behavior and referral patterns ultimately do lead to fewer non-Hispanic Black patients and patients with public insurance being initially evaluated for concussion in SC, a critical setting to ensure equity in concussion care in light of the important advances in early concussion management implemented by specialists.^4,7^

Beyond the sociodemographic differences noted, an important finding of our study is the significant difference in COI by clinical setting, which represents the first work to describe such differences. The COI was developed as a tool to quantitatively identify the availability of resources critical to the healthy development of children at the neighborhood level^33^ and represents a validated measure that allows a more precise understanding of potential disparities beyond the proxies of race and ethnicity. Each of the domains of the COI represent areas important to the recovery of children with concussion and may identify aspects that are modifiable to improve equity. For example, the indicators within the health and environment domain include features such as access to green space and neighborhood walkability.^16^ In light of recent findings that early aerobic exercise helps expedite concussion recovery,^4,5^ a lower score in the health and environment domain could be detrimental to recovery. Stark differences exist in the education subdomain for patients first seeking concussion care in the specialty care setting and the ED (a scaled *z* score of 22 of 100 for pediatric patients in the ED vs 88 of 100 for patients in specialty care). School reentry with support is a critical component of concussion recovery,^34^ with recent data indicating that children who are able to return to school more expeditiously have shortened recovery times.^35^ Pediatric patients with concussion often need accommodations to return to the school environment after concussion,^8^ and schools with fewer resources are likely to have more difficulty implementing such accommodations. It is important to note that COI itself can serve as a proxy for race^36,37^—in light of historically and systemically racist segregation practices, such as neighborhood red-lining^38^—further underscoring the intricate interplay between race, opportunity, and health outcomes for children in the United States.

Interestingly, we found that while the previously mentioned disparities persisted throughout the COVID-19 pandemic periods, they were less pronounced during the first pandemic year. This is in contrast to findings of other pediatric disease processes, including mental health and preventative care.^26,39^ This may be due to a lack of availability of outpatient care resources, leading those with more opportunity to turn to EDs as a point of entry, or due to shifting injury mechanisms and severity,^40^ given a reduction in organized sport during the early portion of the pandemic. Further work investigating the impact of COVID-19 on care-seeking behaviors for pediatric concussion is required.

Regardless of the ultimate etiology of the differences found in our study, our results demonstrate that those at highest risk for disparities in concussion care and recovery are seeking care in the ED, a setting least equipped to provide individualized targeted concussion guidance. As concussion care moves away from a one-size-fits-all approach to a more targeted active management approach,^41^ more time is likely required to determine individualized recovery strategies. Prior work has found that fewer than half of children who receive a concussion diagnosis in the ED ever receive any additional care from another clinician after being discharged,^42^ highlighting the key role that ED clinicians play in providing comprehensive anticipatory guidance for concussion. Electronic clinical decision support (eCDS) may serve a role in supporting pediatric emergency medicine clinicians in this area, as prior studies have found the utilization of eCDS can help facilitate the provision of concussion-specific care in the ED^31^ and that pediatric emergency clinicians find them helpful in improving care.^43,44^ In addition, in recent years, a validated score for risk stratifying pediatric patients with concussion from the ED setting (the 5P score) has been derived and validated across multiple studies^45,46^ and shown to be implementable across both academic and community EDs.^47^ Beyond extending clinician training, our results also underscore the importance of establishing resources at the community level and enhancing training for all those who may interact with children with concussions.^34^ Regardless of where a child with concussion seeks initial care, they must return to school and often to sports and other organized physical activities. Determining how these community settings can serve to further strengthen the medical home and reinforce the anticipatory guidance provided at their point of entry into the health care system for concussion is a key area of future research.

### Limitations

There are several limitations to our study. We relied on EHR data for our race and ethnicity characterizations; previous work has identified the limitations and potential inaccuracies of this approach.^48,49^ However, given the size of our sample, we do not possess an alternative method to assess these characteristics. Next, our use of COI, while a strength in assessing neighborhood-level features of opportunity at the census tract level, does not account for individual measures of opportunity or vulnerability. Specifically, while the COI version 3.0 includes data through 2021, it may not account for rapid gentrification of specific neighborhoods nor identify families with higher socioeconomic status living in historically lower SES neighborhoods,^50^ both of which may impact care seeking behaviors. As an administrative database, our data may have been at risk for inaccurate documentation or coding (the database has not undergone formal validity testing), and certain variables were not able to be abstracted from all health care settings in our data, including injury mechanism and treatment plans. Our cohort only included those who received a concussion diagnosis; as noted previously, disparities in the assignment of a concussion diagnosis exist by sociodemographic characteristics.^12^ Furthermore, our data were obtained from a single regional health care system in the northeastern United States. Therefore, our findings may not be generalizable to other geographic locations. However, previous work has found that our geographic model (ED located in an urban area and SC primarily in suburban areas) exists in multiple pediatric health systems for conditions beyond concussions, and the disparities that result from this distribution of clinical care resources likely already exist across other health care networks.^51,52,53^

## Conclusions

In this cross-sectional study, we found that among pediatric patients seeking initial concussion care within a regional tertiary care network, those seen in the ED were more likely to be non-Hispanic Black, have public insurance, and live in areas with lower overall child opportunity compared with those cared for in PC and SC settings. This highlights the importance of providing education and training for pediatric emergency medicine clinicians as well as enhancing neighborhood- and community-level resources for pediatric patients with concussion.

## Notes

Supplement 1. **eTable.** Characteristics of Initial Visit by Presenting Location, Stratified by Time Period as Related to the COVID-19 Pandemic

Supplement 2.
Data Sharing Statement
