# Supplementary material for: Community and Patient Features and Health Care Point of Entry for Pediatric Concussion
Source: JAMA Netw Open. 2024 Oct 30;7(10):e2442332. doi: 10.1001/jamanetworkopen.2024.42332 (PMC11525599; doi:10.1001/jamanetworkopen.2024.42332)
Supplement: Supplement 1. — eTable. Characteristics of Initial Visit by Presenting Location, Stratified by Time Period as Related to the COVID-19 Pandemic [file jamanetwopen-e2442332-s001.pdf]

Supplemental Online Content

Corwin DJ, Fedonni D, McDonald CC, et al. Community and patient features and health care point of entry for pediatric concussion. *JAMA Netw Open.* 2024;7(10):e2442332. doi:10.1001/jamanetworkopen.2024.42332

eTable. Characteristics of Initial Visit by Presenting Location, Stratified by Time Period as Related to the COVID-19 Pandemic

This supplemental material has been provided by the authors to give readers additional information about their work.

© 2024 Corwin DJ et al. *JAMA Network Open.*

eTable. Characteristics of Initial Visit by Presenting Location, Stratified by Time Period as Related to the COVID-19 Pandemic

	**Pre COVID (1/1/2017-3/15/2020)**			**Early COVID (3/16/2020-3/15/2021)**			**Late COVID (3/16/2021-8/4/2023)**			
	**ED,** N = 1,766	**PC,** N = 5,557	**SC,** N = 2,618	**ED,** N = 219	**PC,** N = 441	**SC,** N = 241	**ED,** N = 984	**PC,** N = 2,419	**SC,** N = 1,386	**p-value**
**Characteristic**	n (%) or median (interquartile range)									
Age										**<0.001**
0-4.99 years	144 (8.2%)	85 (1.5%)	19 (0.7%)	44 (20.1%)	14 (3.2%)	6 (2.5%)	99 (10.1%)	41 (1.7%)	19 (1.4%)	
5-7.99 years	215 (12.2%)	288 (5.2%)	67 (2.6%)	32 (14.6%)	37 (8.4%)	12 (5%)	123 (12.5%)	160 (6.6%)	57 (4.1%)	
8-12.99 years	639 (36.2%)	1,905 (34.3%)	630 (24.1%)	37 (16.9%)	132 (29.9%)	44 (18.3%)	342 (34.8%)	741 (30.6%)	304 (21.9%)	
13-17.99 years	768 (43.5%)	3,279 (59%)	1,902 (72.7%)	106 (48.4%)	258 (58.5%)	179 (74.3%)	420 (42.7%)	1,477 (61.1%)	1,006 (72.6%)	
Biological Sex										**<0.001**
Female	736 (41.7%)	2,762 (49.7%)	1,417 (54.1%)	105 (47.9%)	221 (50.1%)	131 (54.4%)	437 (44.4%)	1,165 (48.2%)	778 (56.1%)	
Male	1,030 (58.3%)	2,795 (50.3%)	1,201 (45.9%)	114 (52.1%)	220 (49.9%)	110 (45.6%)	547 (55.6%)	1,254 (51.8%)	608 (43.9%)	
Race/Ethnicity										**<0.001**
Hispanic	136 (7.7%)	332 (6%)	150 (5.7%)	19 (8.7%)	17 (3.9%)	13 (5.4%)	92 (9.3%)	185 (7.6%)	111 (8.0%)	
Non-Hispanic Black	923 (52.3%)	676 (12.2%)	210 (8%)	84 (38.4%)	43 (9.8%)	27 (11.2%)	478 (48.6%)	293 (12.1%)	131 (9.5%)	
Non-Hispanic White	561 (31.8%)	3,946 (71%)	1,966 (75.1%)	87 (39.7%)	343 (77.8%)	168 (69.7%)	296 (30.1%)	1,584 (65.5%)	936 (67.5%)	
Other[1]	146 (8.3%)	603 (10.9%)	292 (11.2%)	29 (13.2%)	38 (8.6%)	33 (13.7%)	118 (12%)	357 (14.8%)	208 (15%)	
Insurance Type										**<0.001**
Private	854 (48.4%)	4,527 (81.5%)	2,207 (84.3%)	118 (53.9%)	362 (82.1%)	200 (83%)	435 (44.2%)	1,904 (78.7%)	1,155 (83.3%)	
Medicaid	912 (51.6%)	1,030 (18.5%)	411 (15.7%)	101 (46.1%)	79 (17.9%)	41 (17%)	549 (55.8%)	515 (21.3%)	231 (16.7%)	
Median income code, dollars	53,750 (37,434, 77,091)	97,515 (76,854, 112,373)	97,008 (77,096, 116,895)	58,782 (37,434, 86,979)	97,515 (78,309, 112,373)	99,130 (79,013, 117,544)	54,052 (36,884, 77,091)	97,515 (76,973, 112,373)	97,362 (75,938, 116,895)	**<0.001**

© 2024 Corwin DJ et al. *JAMA Network Open.*

	Pre COVID (1/1/2017-3/15/2020)			Early COVID (3/16/2020-3/15/2021)			Late COVID (3/16/2021-8/4/2023)			p-value
	**ED,** N = 1,766	**PC**, N = 5,557	**SC**, N = 2,618	**ED**, N = 219	**PC**, N = 441	**SC**, N = 241	**ED**, N = 984	**PC**, N = 2,419	**SC**, N = 1,386	
% of residents in zip code with bachelor's degree or higher	28 (18, 42)	49 33, 60)	49 (33, 60)	31 (19, 52)	49 (33, 62)	51 (33, 60)	29 (18, 42)	51 (33, 62)	47 (32, 60)	**<0.001**
Median COI: Overall[2]	24 (7, 67)	86 (67, 94)	87 (69, 95)	44 (14, 82)	89 (72, 95)	87 (69, 96)	35 (12, 73)	89 (70, 96)	87 (69, 96)	**<0.001**
Median COI: Educational[2]	21 (8, 66)	88 (70, 96)	89 (72, 96)	36 (10, 82.5)	90 (74, 96)	89 (71, 96)	26 (7, 67)	89 (69, 96)	87 (65, 96)	**<0.001**
Median COI: Health/ Environmental[2]	49 (30, 71)	76 (56, 89)	78 (60, 89)	68 (51, 88)	83 (67, 94)	84 (67, 94)	69 (49, 86)	85 (69, 95)	86 (70, 94)	**<0.001**
Median COI: Social/Economic[2]	24 (6, 66)	84 (65, 93)	84 (66, 93)	43 (11, 80.5)	86 (70, 94)	85 (66, 94)	34 (10, 73.3)	87 (68, 94)	86 (68, 95)	**<0.001**

COI = child opportunity index; ED = emergency department; PC = primary care; SC = specialty care

[1] "Other" includes American Indian/Alaskan Native, Asian, Native Hawaiian or Other Pacific Islander, as well as multiracial children, children for whom race as not reported, and those who selected "other race" during registration

[2]Child Opportunity Index is a scaled Z-score which ranges from 0-100, where 0 represent least opportunity and 100 represents most opportunity

© 2024 Corwin DJ et al. *JAMA Network Open.*
