# Supplementary material for: Community and Patient Features and Health Care Point of Entry for Pediatric Concussion
Source: JAMA Netw Open. 2024 Oct 30;7(10):e2442332. doi: 10.1001/jamanetworkopen.2024.42332 (PMC11525599; doi:10.1001/jamanetworkopen.2024.42332)
Supplement: Supplement 2. — Data Sharing Statement [file jamanetwopen-e2442332-s002.pdf]

Data Sharing Statement

Corwin. Community and Patient Features and Health Care Point of Entry for Pediatric Concussion. *JAMA Netw Open*. Published October 30, 2024.
doi:10.1001/jamanetworkopen.2024.42332

Data
Data available: Yes
Data types: Data dictionary
How to access data: corwind@chop.edu
When available: With publication

Supporting Documents
Document types: None

Additional Information
Who can access the data: Researchers whose proposed use of the data has been approved
Types of analyses: For any purpose
Mechanisms of data availability: With a signed data access agreement
